# Are topological explanations really free of mechanisms?

**DOI:** 10.1007/s12064-020-00336-0

**Published:** 2021-01-11

**Authors:** Xin Zhang

**Affiliations:** Dalian University of Technology, Dalian, China

**Keywords:** Topological explanation, Non-mechanistic explanation, Mechanistic explanation, Mechanism, Explanatory relevance

## Abstract

Topological explanations in biology have been largely assumed to be free of mechanisms. However, by examining two classic topological explanations in the philosophical literature, this article has identified mechanisms in the corrected and complete formulations of both explanations. This constitutes the major work of this article. The minor work of this article is to address a follow-up question: given that these two topological explanations contain mechanisms, would this significantly blur the widely assumed boundary between topological and mechanistic explanations? My answer to this question is negative and the argument I have developed is that although these two topological explanations contain mechanisms, these mechanisms are explanatorily irrelevant to the target properties, which is in stark contrast to the situation in mechanistic explanations.

## Introduction

Mechanistic explanations have proved to be the most prevailing type of explanation in biology. Nevertheless, the existence and prominence of non-mechanistic explanations in biology have long been recognized (Woodward 2013). Recent years see a surge of philosophical discussions on non-mechanistic explanations in various biosciences, including but not exclusive to ecology (Huneman 2010), evolutionary biology (Baker 2009; Huneman 2018b), neuroscience (Kostić 2018b), systems biology (Green et al. 2018), immunology (Jones 2014) and network medicine (Darrason 2018). In these discussions, different types of non-mechanistic explanation have been identified, for example, dynamical model explanation (Ross 2015), minimal model explanation (Batterman and Rice 2014) and optimality explanation (Rice 2015). Among them, one particular type of non-mechanistic explanation, i.e., topological explanation, has received special attention and this article mainly revolves around it.

What is topological explanation? Presently, a consensus has been reached that topological explanation is a process where a system’s one physical property is explained solely by the system’s one or a few topological properties (Huneman 2010, 2018b, c; Kostić 2018a, 2019a, b).^1^ Notwithstanding, there are two major points of disagreement over the interpretation of this delineation.

The first disagreement concerns the interpretation of “is explained by” in the delineation above. Huneman (2010, 2018a, c) has provided an entailment interpretation of this expression. That is, to say a physical property is explained by a topological property amounts to saying that the physical property, which can be framed as a mathematical fact, is entailed by the topological property. On the other hand, Kostić (2019a, b) has offered a counterfactual interpretation of the expression. That is, to say a physical property is explained by a topological property means that had the topological property not obtained, then the physical property would not have obtained; or, had the topological property been different, then the physical property would have been differed.

Despite the disagreement, it seems that Huneman in some places also admits a counterfactual interpretation. For instance, at one place where Huneman (2018c) elaborates on structural explanation, of which topological explanation has been considered as a kind, he has explicitly adopted a counterfactual interpretation of the expression above, i.e., “…the mathematical property about volume to surface ratio explains why two applications of the law of heat loss to two animals differing in their average size (other things being equal) yield different results. Simply put, has this law not obtained, there would be no Bergmann’s rule.”

This is understandable because as pointed out by one anonymous reviewer, the opposition between the entailment interpretation and the counterfactual interpretation can be dissolved if we realize that they are characterizations from different angles. The entailment interpretation emphasizes that the explaining process takes the form of entailment, but this does not forbid that this entailment be based on certain counterfactual relation between physical and topological properties. On the other hand, the counterfactual interpretation highlights that the explanatory relation between physical and topological properties takes the form of counterfactuals, but this does forbid that the explaining process can take the form of entailment. In view of this, no choice needs to be made between these two interpretations.

In spite of this, I will make a subtle change to the counterfactual interpretation. Kostić’s counterfactual interpretation is of the classic form developed by Lewis (1973, 2001), but I will adopt a more interventionist-like, though still counterfactual, form. That is, I treat as variables both the topology of a system (variable T) and the obtaining state of the target physical property (variable P). Then, I check whether P’s value or distribution will change when I change T’s value from its actual one to a counterfactual one. If the answer is positive, the target physical property is explained by the actual topology of the system. I call this form interventionist-like due to its similarity to the interventionist causal theory: this form treats as variables both the topology of a system and the obtaining state of the target physical property, while the interventionist causal theory treats as variables both causes and effects. Nevertheless, this form shall not be confounded with the interventionist causal theory because in this form I have not imported the important notion of “intervention.” I purposely do so because I do not intend this form to check for causal explanatory relations in particular, but explanatory relations in general.

The reason why I adopt the interventionist-like form consists in that, as pointed out by one anonymous reviewer, the classic form by Lewis suffers from the fact that it is sometimes difficult to agree on the metrics across possible worlds and hence difficult to decide what the closest possible world is like. For instance, suppose a graph is scale-free, then in the closest possible world where this scale-freeness had not obtained or the graph had had certain topology other than scale-freeness, it is uncertain what topology this graph would have assumed. The interventionist-like form, on the other hand, circumvents this problem because according to it, we need to first change the topology from the actual one to a specified counterfactual one, and then check whether the target physical property still obtains in this counterfactual scenario. Therefore, we are always certain about the topology of the target system in the counterfactual world.

The second point of disagreement centers on what topological properties really are. Two major conceptions of topological property can be found in the literature, an inclusive one by Huneman (2010, 2018c) and an exclusive one by Kostić (2019a). Huneman’s definition of topological property is quite akin to the definition of topological property in the mathematical field of topology (mathematical topological property hereafter). First, we represent a system S as structure S’ in a topological space E. Then, topological properties are just those of S’ that stay invariant under continuous transformations and hence determine equivalence classes between all structures homotopic to S’ (Huneman 2010, 2018c). This definition is inclusive because on it, topological properties cover not only graph-theoretical properties (e.g., connectedness, small-worldness, scale-freeness, modularity), which are the most commonly observed type of topological property in the literature, but also many other types of structural property, i.e., properties concerning the structure of a system or a certain representation of the system. For instance, on the inclusive conception, the flatness and sharpness of a genotype’s neighborhood in a fitness landscape are topological properties (Huneman 2010). Meanwhile, on the inclusive conception, the many-to-one feature of the mapping from triplets of nucleotides to amino acids is also a topological property (Huneman 2010).

In contrast, Kostić’s conception of topological property is more exclusive, because on it, topological properties refer exclusively to mathematical properties of connectivity patterns in complex networks (Kostić 2019a). Consequently, the only type of structural properties qualified as topological properties are graph-theoretical properties.

Both conceptions of topological property have their merits. For instance, unlike the exclusive conception, the inclusive conception is in the same vein as the conception of mathematical topological property and hence can preclude certain nomenclature confusion. In fact, under the exclusive conception, it is best to abandon the term “topological properties” altogether and call these properties graph-theoretical properties directly. Also, as suggested by one anonymous reviewer, on the exclusive conception, it is more accurate to rename topological explanations as graph-theoretical explanations. In spite of this, the merit of the exclusive conception is also hard to dismiss. That is, it accommodates the current situation where the majority of topological explanations under study are characterized by graph-theoretical properties, instead of mathematical topological properties in general. As for my purpose here, I see no need to take a side between these two conceptions, because the two topological explanations I am going to examine both feature graph-theoretical properties and hence are in accordance with both conceptions. Nevertheless, I do sympathize with the former idea that it is more accurate to rename topological explanations as graph-theoretical explanations if we decide to focus exclusively on graph-theoretical properties of target systems.

With this elaboration on the concept of topological explanation in place, I now present the subject matter of this article. From the very beginning of the recent surge of philosophical discussions on topological explanation, it has been put in contrast to mechanistic explanation (Darrason 2018; Huneman 2010; Kostić 2018a). One major grounding for this contrasting relation consists in that topological explanations are devoid of mechanisms (Huneman 2010). Here, “mechanisms” has been used in the sense advocated by new mechanists, that is, they are organized collections of components and their activities (Craver and Bechtel 2007; Machamer et al. 2000). More specifically, for most mechanisms, these activities of components form a temporal sequence where neighboring steps are causally connected.

For instance, according to Huneman (2010, 2018a), topological explanations proceed by entailing the explananda, which can be formulated as mathematical facts, from topological properties, which are mathematical properties. Therefore, a topological explanation is in essence a mathematical entailment and therefore apparently excludes mechanisms. On the other hand, according to Kostić (2019b), “the topological explanation has a structure of counterfactual that describes a mathematical dependency between a set of topological properties and a network representation of a real-world system.” Admittedly, mathematical dependences can be causal or constitutive (Glennan 2017). Nevertheless, those that appear in Kostić’s conception of topological explanation are neither (Kostić 2019b). Consequently, on the conception given by Kostić, topological explanation also excludes mechanisms, because mechanisms typically exhibit both causal and constitutive dependences.

It is noteworthy that although topological explanations have been considered to be devoid of mechanisms, this does not forbid topological explanations be integrated with mechanisms to achieve certain explanatory goals. For instance, Huneman (2018a) has highlighted two types of such integration. The first type can be labeled as the constraining type and it consists in that for a system whose dynamics is partially constrained by its topology, a full explanation of its dynamics must take into account both its topology and its mechanisms. The second type can be labeled as the inter-level type and it pertains to explanations that take into account multiple levels of the target system (here levels are delimited according to part-whole relations). That is, for an explanation of this kind, the part of it that relates to one level can be of a mechanistic nature, while the part of it that relates to another level can be of a topological nature.

Such hybrid types of topological-mechanistic explanation are sometimes also referred to as topological explanations. Given the fact that they contain mechanisms, it is noteworthy that by asserting topological explanations to be devoid of mechanisms (mechanism-free assumption hereafter), philosophers are referring exclusively to pure topological explanations, a convention that I will follow throughout this article.

The major work of this article is to argue against the mechanism-free assumption above. I will examine two classic topological explanations retrieved from the philosophical literature and demonstrate that mechanisms can be detected in their corrected and complete formulations. This work will be carried out in Sect. 2.

The minor work of this article is to address a follow-up question regarding the major work above: if the two topological explanations do contain mechanisms, would this significantly blur the widely assumed boundary between topological and mechanistic explanations? I suggest the answer to be negative and the reason is that the mechanisms in those two topological explanations are explanatorily irrelevant to the target phenomena. This work will be carried out in Sect. 3. Section 4 concludes the article.

## Mechanisms exist in topological explanations

As mentioned earlier, topological explanations have been largely assumed to be free of mechanisms. However, two topological explanations will be examined in this section and it will be argued that contrary to the mechanism-free assumption, both topological explanations, in their corrected and complete formulations, contain mechanisms.

### An ecological case

The first case I am going to examine is one raised by Huneman (2010), and it concerns the stability of a certain ecological community. Before delving into this case, it is necessary to point out that, as noted by Huneman himself, ecological stability historically has a variety of measures and the one eventually adopted by Huneman is the stability of the frequencies of species within an ecological community. That is, an ecological community is stable if upon perturbations (e.g., extinction of its own species or invasion by other species), the frequencies of species within the community tend to remain largely unchanged.

The target ecological community (note as C hereafter) Huneman has picked is a hypothetical one with two hub species and 464 non-hub species. Here, hub species are those that correspond to densely connected nodes in the food web, while non-hub species are those that correspond to sparsely connected nodes. Huneman suggests that this community is stable in face of random extinction events and has provided a topological explanation for it. Suppose a random extinction event happens to one arbitrary species in the community under focus. This amounts to the removal of an arbitrary node N in the corresponding food web. Since the food web has 464 non-hubs and only two hubs, the chance is that N is much more likely to be a non-hub. Removal of a non-hub, according to Huneman, would not alter the whole structure of the food web, because non-hubs by definition are sparsely connected. From this, Huneman directly derives the stability of the target community.

This explanation accords well with my earlier delineation of topological explanation, that is, it explains the community’s stability solely by appealing to the graph-theoretical property of “two hubs and 464 non-hubs.” To see this, if we treat the food web’s topology as a variable and change its value from “two hubs and 464 non-hubs” to “464-hubs and two non-hubs,” then the community becomes highly unstable in face of random extinction events, because in this counterfactual scenario, a random extinction event is much more likely to fall upon a hub-species and this would result in significant changes in the structure of the food web. Consequently, if we treat the community’s obtaining state of stability as a variable, its value changes for “obtain” to “not obtain” upon the former change of topology.

Prima facie, no mechanisms exist in the topological explanation above. Nevertheless, this explanation is mistaken in three places. First, there is no guarantee that removal of a non-hub would not alter the structure of a graph. Take the conception of bridge as an example. “An edge that joins two nodes A and B in a graph is called a bridge if deleting the edge would cause A and B to lie in two different components.” Here, a component refers to “a subset of the nodes such that (i) every node in the subset has a path to every other and (ii) the subset is not part of some larger set with the property that every node can reach every other” (Easley and Kleinberg 2010). Suppose a non-hub has a bridge as one of its edges, then removal of this non-hub would apparently elicit a profound change in the structure of the graph: our action divides one big component into two small sub-components.

Second, the topological explanation does not explicitly take secondary extinctions into account. Even though we grant that the removal of a non-hub will not by itself alter the structure of the graph, the structure can still be subject to significant changes due to secondary extinctions triggered by the initial extinction event (Dunne et al. 2002). For instance, if the removed non-hub represents a basal species, which is possible because such species tend to have low degrees, then a considerable amount of secondary extinctions are to be expected because such species constitute the energy foundation for the community. Or, if the removed non-hub represents a species that has only one specialist predator and this predator has multiple specialist predators, then apparently the scale of secondary extinctions is also likely to be large (Dunne et al. 2002).

Third, we cannot derive the stability of a community solely from the stability of its structure, because the dynamics of a network is determined not only by its structure of connection, but also by the strengths of these connections (or edges). For instance, depending on the strengths of edges, gene regulatory circuits with the same structure can exhibit divergent dynamical regimes (Jaeger 2018). Therefore, even though we grant that the structure of the food web is not altered by the removal of a non-hub, the dynamics is still possible to undergo a significant change, because the strengths of several edges might be profoundly altered by the removal. For instance, if the non-hub represents a species S_1_ that is the chief prey of species S_2_, then the strengths of edges between S_2_ and its other preys tend to increase significantly after the removal of S_1_, because now S_2_ has to compensate by preying more on its other preys. Once this change of edge strengths brings about a dramatic change in the network dynamics, e.g., change of attractors, the frequencies of species would also tend to experience a dramatic change. Consequently, although the network structure is largely preserved in these scenarios, the network is nevertheless unstable.

To note, as pointed by one anonymous reviewer, both Huneman and I explicitly consider an unweighted graph and therefore my third critique regarding edge weights is dispensable within the scope of this article. Nevertheless, within a larger scope, this critique is noteworthy because it reflects a general neglect of weighted graphs in current philosophical discussion on topological explanations. And this is the reason I preserve the critique here.

**Fig. 1:**
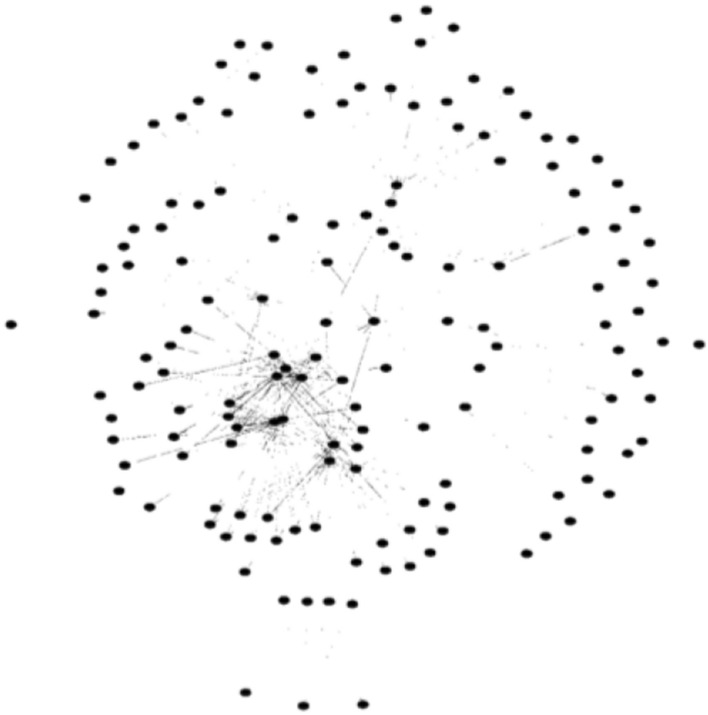
The food web Huneman (2010) has provided as an approximation to the target ecological community

In view of these critiques, I will attempt to raise a new topological explanation. To this end, as rightly noted by an anonymous reviewer, we first need to add more information into the case. To recall, the only information provided to us in the original paper by Huneman is that the food web of C is made up of two hubs and 464 non-hubs. As indicated by the first two critiques above, this piece of information alone does not guarantee the stability of C. Therefore, more information is called for. But what information? In the original paper by Huneman, he has provided a picture that depicts the food web of C (Fig. 1). Admittedly, this picture is of a rather low resolution and, as rightly noted by one anonymous review, it is of a purely illustrative nature. Nevertheless, I suggest that the graph in this picture actually has such a feature that if we add it into the original case, the stability of C can be guaranteed. This feature consists in that among the parts in the picture that can be readily identified, a considerable portion of non-hubs connect exclusively to hubs.

With this additional information in place, I will develop a new topological explanation as below. First, the same as in the original explanation, a random extinction event is very likely to happen to a non-hub species. Second, according to the information we have just added, this non-hub is very likely to connect exclusively to hubs. This feature, for the reason that will be given immediately, guarantees that the extent of secondary effects due to the extinction of this non-hub is likely to be moderate. For illustration, suppose the hub-species is a predator and all species it connects to are its preys. Then, it is highly probable that the hub-species can compensate the extinction of the non-hub by preying more heavily on its other preys. Besides, this compensation tends not to prominently alter the frequencies of these other preys, because the predator is a hub-species and hence has many different preys to share the load of compensation. Consequently, the extent of secondary effects due to the initial extinction event is likely to be moderate, which indicates that the target community is stable against random extinction events.

In contrast to the original explanation, this new explanation contains a mechanism. To recall, the structure of the new explanation consists in that (1) a non-hub species goes extinct, (2) this extinction affects the frequency of a hub species directly connected to this non-hub, and (3) this change of frequency in turn affects the frequencies of other non-hubs connected to this hub species. This structure, when combined with the two graph-theoretical properties aforementioned (i.e., first, that the food web contains two hubs and 464 non-hubs; second, that a considerable portion of non-hubs connect exclusively to hubs.), derives the stability of the target system. I suggest the (1) → (2) → (3) structure is a mechanism because it is an organized collection of components and their activities: the components are hub and non-hub species in the structure and the activities are the extinction or change of frequencies of these species. As stipulated earlier, this is exactly the conception of mechanism adopted by this article. Moreover, in the structure above, the activities of components form a temporal sequence where neighboring steps are causally connected. As aforementioned, this is exactly the form that most mechanisms take. Consequently, I conclude the discussion of this case by claiming that the original topological explanation is mistaken and in one corrected formulation of the original topological explanation, there exists a mechanism.

### A neuroscientific case

Now let us turn our attention to another topological explanation. It is adopted from Kostić (2019a) and relates to the global controllability of brain. The brain is a dynamical system, and its states can be represented by a state vector *X* = (*x*_1_,…, *x*_*n*_). Here, the brain is divided into n regions and *x*_*n*_ represents the state of the region labeled by n. The global controllability of the brain refers to the phenomenon that the brain is capable of making efficient transition from one value of *X* to the another, which has been largely ascribed to the fact that the energy cost of such transitions is relatively low.

To make sense of this controllability, a topological explanation has been provided (Kostić 2019a). First, a graph is drawn where nodes represent the total n brain regions and edges represent white matter tracts in between them. Then, this graph is identified as a small-world network. A small-world network is a graph with low average path length and high clustering coefficient, where average path length measures the typical “distance” between nodes and clustering coefficient captures the typical cliquishness around a node (Watts and Strogatz 1998). This small-worldness indicates that for two arbitrary nodes in the graph, it tends to take a minimal number of edges to go from one node to the other. From this, it has been derived that the energy requisite to change the value of *X* from one to another also tends to be minimal. Therefore, the brain is capable of making efficient transition from one value of *X* to another and hence globally controllable.

This explanation fits well with the depiction of topological explanation adopted by this article, because it explains the target phenomenon solely by appealing to the graph-theoretical property of small-worldness. For illustration, if we treat the topology of the brain region graph as a variable and change its value from “small-worldness” to “high average path length and low clustering coefficient,” then for two arbitrary nodes in the graph, it tends to take a maximal number of edges to go from one node to the other. According to the logic of the previous topological explanation, this indicates that the energy requisite to change the value of *X* from one to the other also tends to be maximal. Therefore, the brain is incapable of making efficient transition from one value of X to the other and hence loses its global controllability. Consequently, if we treat the brain’s obtaining state of global controllability as a variable, its value changes from “obtain” to “not obtain” upon the former change in topology.

On the first sight, the topological explanation above does not contain any mechanism. However, this explanation is incomplete in the following sense. To recall, the explanation has made the derivation as below: (A) for two arbitrary nodes in the graph, it tends to take a minimal number of edges to go from one node to another → (B) the energy requisite to change the value of X from one to the other tends to be minimal. But what guarantees this derivation? I find no existing laws or regularities that directly support this step of derivation. In fact, the subject matter in (A) is the walking between nodes, while the subject matter in (B) is the change of the value of *X*. To make the derivation understandable, we need to add information about the relationship between these two subject matters. That is, how is the change of value of *X* related to the walking between nodes. On the basis of this, I suggest the original topological explanation is incomplete.

Before taking steps to complement the original explanation, it is noteworthy that as pointed out by one anonymous reviewer, Kostić (2019a) has already in the very same paper noted the incompleteness of the explanation and thereafter provided additional steps to complement it. Nevertheless, this does not render my work here redundant because the point of incompleteness noted by Kostić is different from that by me. According to Kostić, the original explanation might be deemed as incomplete in scenarios where we are not satisfied by simply knowing the graph has a particular topology, but raise the further inquiry of “how and why the system arrived at having certain topological properties.” In these scenarios, as rightly suggested by Kostić, we need to add to the original topological explanation a mechanistic explanation regarding the formation of the particular topology (small-worldness in this case). However, this is not the point of incompleteness identified by my former discussion. To recall, according to that discussion, the original topological explanation is incomplete not because we might have the further inquiry over the mechanism that gives rise to the topology, but because the original topological explanation itself is incomplete in its own logical structure, i.e., the derivation from (A) → (B) is not guaranteed by known laws or regularities. Consequently, these are two different points of incompleteness and my work here is hence not redundant.

Now let us return to the point of incompleteness identified in my former discussion. I suggest that this point can be fixed by adding the following part into the original explanation:

For simplicity, suppose the transition of *X* = (*x*_1_,…, *x*_1_) from one value to another is caused by one single regulatory electrical signal originated from a certain brain region. For the effect of this signal to spread to the brain regions whose states (i.e., the value of *x*_*i*_) need to be changed, the signal has to travel along the edges in the brain graph and hence go from one region to another.

After adding this part into the original explanation, the (A) → (B) derivation becomes understandable. (A) states that for two arbitrary brain regions, it tends to take a minimal number of edges to go from one to the other. In view of this, the energy requisite for the signal to reach each of its target regions is minimal. According to the part added earlier, these energies altogether comprise the energy requisite for *X* to change from one value to another. Therefore, the energy requisite to change the value of *X* from one to another is also minimal, which is the content of (B).

After integrating the previous part into the original explanation, we come to a new explanation and as will be argued, this new explanation contains a mechanism. To recall, the main structure of this new explanation is the spread of the signal from its initial brain region to its targeted brain regions. This main structure, when combined with the small-worldness of the graph, indicates the low energy costs of brain state transitions and hence the controllability of the brain. I suggest that this main structure conforms to our earlier depiction of mechanisms, because it is an organized collection of components and their activities: the components here are various brain regions involved in the structure and the activities are the activation or repression of these region in the progression of the original signal. Moreover, in the structure above, the activities of components form a temporal sequence where neighboring steps are causally connected. As aforementioned, this is exactly the form that most mechanisms take. On the basis of this, I argue that in the neuroscientific case, the original topological explanation is incomplete and in one complemented formulation of the original explanation, we detect a mechanism.

Nevertheless, as noted by one anonymous reviewer, it might be argued that my conclusion regarding the neuroscientific case is mistaken, for the following reason. Admittedly, the new explanation in this case does contain certain empirical facts, for instance, we have mentioned signals, brain regions and white tracts in it. However, we only mention these facts because the explanandum is a physical property, that is, if the explanation goes purely at the mathematical level, it will not constitute the explanation of a physical property. Therefore, the real explanatory work goes at the mathematical level, and hence, the mentioning of these empirical facts does not make the explanation mechanistic.

I agree with the content in this critique, but I do not consider it as a threat to my former conclusion, for two reasons. First, the conclusion that the new explanation includes a mechanism is not based on the mere fact that the explanation mentions empirical facts, but based on the observation that these facts constitute an organized collection of components and their activities. Second, that the real explanatory work goes at the mathematical level does not immediately render an explanation non-mechanistic. In scenarios where the mathematical structure does not map onto a certain causal structure, the explanation is indeed non-mechanistic. Some philosophers refer to this type of explanations as distinctively mathematical scientific explanations (Lange 2013, 2017). For instance, if we explain why a mother cannot divide 4 apples evenly among her 3 children by drawing on the mathematical fact that 4 is indivisible by 3, what we have is a non-mechanistic explanation because the mathematical structure in this case (i.e., 4, 3, 4/3) does not depict any causal relation. In contrast, in scenarios where the mathematical structure does map onto a certain causal structure and this structure is indeed integrated into the explanatory process, the explanation is at least partially mechanistic. Philosophers have denoted these explanations as scientific explanations that merely employ mathematics. For instance, if we explain the long-term behavior of a dynamic system by applying dynamic systems theory to a set of dynamical equations, what we have is a mechanistic explanation because this set of equations depicts causal relations among components in the system and these relations are appealed to by the explanatory regime. As to our neuroscientific case, I suggest the mathematical structure, i.e., the graph is more than just an abstraction of the brain structure, but also depicts the causal relations among brain regions: that signals can be spread from one brain region to another through edges. Moreover, to recall, these causal relations are indeed adopted by the new explanation: the main structure of this explanation consists in the spread of the signal from its initial brain region to its targeted brain regions. In view of this, I suggest that although the real explanatory work in the new explanation goes at the mathematical level, this explanation is still partially mechanistic.

## The boundary between topological and mechanistic explanations

By now, we have finished the major work in this article, that is, to point out that contra the common mechanism-free assumption towards topological explanations, we have found mechanisms in the corrected and complete formulations of two classic topological explanations. In this section, we pursue a follow-up question: given that these two topological explanations contain mechanisms, would this significantly blur the widely assumed boundary between topological and mechanistic explanations? For instance, Huneman (2018a) has explicitly urged that topological explanations and mechanistic explanations are distinct in principle and even rejected the idea that two types of explanation are at two poles of a continuum. My answer to the question above is negative and the argument I am going to back up is that although these two topological explanations contain mechanisms, they are explanatorily irrelevant to the explananda, which is in stark contrast to the situation in mechanistic explanations.

Before elaborating on this argument, it is crucial to note that Huneman (2010, 2018a, c) has also argued that mechanisms are explanatorily irrelevant to the explananda of topological explanations. Nevertheless, this argument is different the one I am aiming for here. In Huneman’s argument, the mechanisms do not appear in the formulation of a topological explanation. For instance, suppose a physical property is explained by a topological property. What Huneman (2010) argues for is that even if the physical property causally results from a certain mechanism, this mechanism is not explanatory because conditioned on the obtaining of the topological property, another mechanism is also capable of producing the physical property. In this scenario, these mechanisms do not appear in the topological explanation of the physical property, but exist as independent candidate explanations of the physical property. In contrast, in the argument I am aiming for here, the mechanisms are those that make appearance in the previous two topological explanations. Consequently, the argument I am going to back up here is distinct from that of Huneman.

To back my argument, we need a proper account of explanatory relevance. Several accounts of explanatory relevance are available and a considerable proportion of them have drawn upon the notion of difference-making: A is explanatorily relevant to B if A, in some sense, makes a difference to the obtaining of B (Strevens 2011). The disagreement among various accounts, though, lies at their different interpretations of the exact sense in which A makes a difference to the obtaining of B (Strevens 2011). For instance, on the probabilistic account, A makes difference to the obtaining of B in the sense that A changes the probability of the obtaining of B. On the counterfactual account, the sense in which A makes a difference to B consists in that had A not happened, B would not have happened. While on the interventionist account, A and B are treated as variables. A is explanatorily relevant to B if there exist interventions on A that change the value of B (or the probability distribution of B).

Among these alternatives, I pick the interventionist account of explanatory relevance. The reason is that, to recall, in the very beginning of this article where I try to settle for a proper depiction of topological explanation, I have chosen the interventionist-like interpretation regarding the sense in which a physical property of a system is explained by its topological properties. To keep consistent with this choice, here I adopt the interventionist account of explanatory relevance: a mechanism is explanatorily relevant to the target property if by replacing a certain entity in the mechanism with another we can change the obtaining state of the target property from “obtain” to “not obtain.” Here, the two concerned variables are (1) the occupying entity of a certain position in the mechanism and (2) the obtaining state of the target property. To note, this interventionist account is different from the interventionist causal theory because in it I have not imported the fundamental notion of intervention. I purposely do so because I do not intend it to check for causal explanatory relations in particular, but explanatory relations in general.

With the interventionist account of explanatory relevance in place, let us check whether mechanisms in those two topological explanations are relevant to their explananda. First, let us look at the ecological case. To recall, the mechanism there consists in that (1) a random extinction event happens to one node in the food web and let us note this node as P; (2) P goes extinct and this extinction affects the frequency of a hub species that P connects to and (3) this change of frequency in turn affects the frequencies of other species connected to this hub species. If we change P to another node Q, would this make any difference to the stability of the community? I suggest the answer is negative, because as long as the two graph-theoretical properties in this case (i.e., first, that the food web contains two hubs and 464 non-hubs; second, that a considerable portion of non-hubs connect exclusively to hubs) stay unchanged, Q is still very likely to be a non-hub, and hence, its extinction still tends not to significantly perturb the whole community. Therefore, according to interventionist account of explanatory relevance, this mechanism is irrelevant to the target property.

Next, let us look at the neuroscientific case. To recall, in this case, the mechanism is the spread of the signal from the initial brain region (noted here as R) to the targeted brain regions (noted here as set {*R*_*i*_}). If we change R to another region R’ and/or if we change {*R*_*i*_} to another set {*R*′_*i*_}, would this make any difference to the brain’s global controllability? The answer is also negative, because as long as the brain graph is a small-world one, it still tends to take a minimal number of edges to go from *R*′ to {* R*′_*i*_ }. Therefore, according to interventionist account of explanatory relevance, this mechanism is also irrelevant to the target property.

Taken altogether, although both topological explanations contain mechanisms, these mechanisms are explanatorily irrelevant to their explananda. This feature sets the two topological explanations in sharp contrast to mechanistic explanations because in the latter case, mechanisms are no doubt explanatorily relevant to their explananda, that is, it is always possible to change the occupying entity of a certain position in the mechanism and then make a difference to the target property (Craver 2006). For instance, in the mechanism of transcription, a change of one transcription factor can completely terminate the transcription process. Or, in the mechanism of signal transduction, a change of one ligand receptor can prevent the ligand from binding and hence stop the signal transduction process. Consequently, the existence of mechanisms in the previous two topological explanations does not blur the commonly assumed boundary between topological and mechanistic explanations. To note, one crucial indication of this conclusion is that to determine whether an explanation is topological or mechanistic, we might focus more on what ingredient in the explanation the explanandum is explanatorily dependent on, and less on whether there is a mechanism in the explanation. This indication is in line with Glennan's (2017) approach to distinguish between causal and non-causal explanations in general.

One last caveat, it might be argued that there is a more convenient way to deal with the boundary problem above: the existence of mechanisms in the corrected and complete formulations of those two topological explanations poses no threat to the boundary between topological and mechanistic explanations, because these corrected and complete formulations are no longer pure topological explanations, but hybrids of topological and mechanistic explanations (hybrid explanations hereafter). I suggest this argument is mistaken, for the following reason. The fundamental distinction between a pure topological explanation and a hybrid explanation lies in that for a hybrid explanation, the target property is not explained solely by a certain topological property, but by a combination of that topological property and a certain mechanism. For instance, one major type of hybrid explanations is the inter-level hybrid explanations (Huneman 2018a). Suppose a system *X* is composed of components {*M*_*i*_} and each *M*_*i*_ is in turn composed of sub-components {${N}_{j}^{i}$}. Assume the target property of *X* is mechanistically explained by an organized set of interactions among {*M*_*i*_}, while each property of *M*_*i*_ that appears in this mechanism is topologically explained by graph-theoretical properties of the graph composed of {${N}_{j}^{i}$}. Combining explanations at both levels, we arrive at an inter-level hybrid explanation of P. Apparently, in this explanation P is explained by both the topological properties at the N-level and the mechanism  at  the M-level. In contrast, our previous discussion suggests that in the corrected and complete formulations of the two topological explanations, the involved mechanisms are explanatorily irrelevant and hence the target properties are explained solely by topological properties. Consequently, the corrected and complete formulations are not hybrid explanations but pure topological explanations. Therefore, the previous argument is mistaken.

## Conclusion

Topological explanations have been taken to be free of mechanisms. However, by examining two classic topological explanations in the philosophical literature, one ecological case from Huneman (2010) and one neuroscientific case from Kostić (2019a), I have detected mechanisms in the corrected and complete formulations of both explanations. Therefore, the mechanism-free assumption regarding topological explanations is mistaken. This constitutes the major work of this article.

The minor work of this article deals with a follow-up question: given that these two topological explanations contain mechanisms, would this significantly blur the widely assumed boundary between topological and mechanistic explanations? My answer to this question is negative and the argument I have developed is that although these two explanations contain mechanisms, these mechanisms are explanatorily irrelevant to the target properties, which is in stark contrast to the situation in mechanistic explanations.

## Footnotes

1 More precisely speaking, topological properties are not properties of the target system per se, but properties of certain representation of the target system (e.g., in most cases a graph).
